# Protocol for a Systematic Review on the Effectiveness of Interventions to Reduce Exposure to Occupational Solar UltraViolet Radiation (UVR) Among Outdoor Workers

**DOI:** 10.3389/fpubh.2021.756566

**Published:** 2021-11-11

**Authors:** Alberto Modenese, Tom Loney, Marc Rocholl, Cara Symanzik, Fabriziomaria Gobba, Swen Malte John, Kurt Straif, Marilia Silva Paulo

**Affiliations:** ^1^Department of Biomedical, Metabolic and Neural Sciences, University of Modena & Reggio Emilia, Modena, Italy; ^2^College of Medicine, Mohammed Bin Rashid University of Medicine and Health Sciences, Dubai, United Arab Emirates; ^3^Department of Dermatology, Environmental Medicine and Health Theory, Institute for Health Research and Education, University of Osnabrück, Osnabrück, Germany; ^4^Institute for Interdisciplinary Dermatological Prevention and Rehabilitation (iDerm), University of Osnabrück, Osnabrück, Germany; ^5^Instituto Salud Global (ISGlobal), Barcelona, Spain; ^6^Department of Biology, Boston College, Chestnut Hill, MA, United States; ^7^Institute of Public Health - College of Medicine, United Arab Emirates University, Al Ain, United Arab Emirates

**Keywords:** intervention, occupational exposure, outdoor worker, skin cancer, sun-safety, UltraViolet Radiation, workplace-based, systematic review protocol

## Abstract

**Background:** Solar UltraViolet Radiation (UVR) is considered the most relevant occupational carcinogenic exposure in terms of the number of workers exposed (i.e., outdoor workers) and UVR-induced skin cancers are among the most frequent types of occupational cancers worldwide. This review aims to collect and evaluate all the available preventive interventions conducted on outdoor workers to reduce their solar UVR related risk, with the final purpose of reducing the burden of occupational skin cancers for outdoor workers.

**Methods:** We will search the following databases for peer-reviewed original research published: MEDLINE (through PubMed), Scopus, and EMBASE. We will include only interventional studies, both randomized and non-randomized, with an adequate comparison group, therefore excluding cross-sectional studies, as well as case-reports/series, reviews, and letters/comments. The systematic review will adhere to the “Preferred Reporting Items for Systematic reviews and Meta-Analyses” (PRISMA) guidelines for reporting systematic reviews. After the literature search, studies to be included will be independently reviewed by two Authors, first based on title and abstract, then based on the full text, according to the inclusion criteria. Conflicts will be solved by a third Author. Two authors will independently extract the required data from included studies and perform quality assessment according to the relevant domain for Risk of Bias assessment proposed by the Cochrane collaboration group. In case of sufficient homogeneity of interventions and outcomes evaluated, results from subgroups of studies will be pooled together in a meta-analysis.

**Discussion:** Following the principles for the evaluation of interventions for cancer prevention established by the International Agency for Research on Cancer, this systematic review will investigate the effectiveness of the interventions, and consequently it will provide reliable indications for the actual reduction of skin cancer incidence in outdoor workers.

## Introduction

### Occupational Solar Ultraviolet Exposure and Skin Cancers

Solar UltraViolet Radiation (UVR) is the most relevant occupational carcinogenic exposure in terms of the number of workers exposed (i.e., outdoor workers) (1–3) and it is the most important risk factor for the development of non-melanoma skin cancer (NMSC; also referred to as keratinocyte carcinoma—KC) (4) and malignant melanoma (MM) (5). The rising incidence of skin cancer over the years has made it a significant public health issue. In 2017, there were more than 3,00,000 cases of MM and about 7.7 million new cases of KC worldwide-−5.9 million due to basal cell (BCC) and 1.8 million due to squamous cell carcinoma (SCC) (6). The *International Agency for Research on Cancer* (IARC) legitimately classified UVR as carcinogenic to humans (Group 1) (7). Especially outdoor workers (e.g., construction workers, fishermen, and farmers) are exposed to high levels of UVR as they spend major parts of their working hours outside (8). Therefore, outdoor workers are at increased risk for developing (occupational) skin cancer. Epidemiologic data show the strikingly high occurrence of both BCC and SCC among outdoor workers after years of cumulative sunlight exposure and clearly demonstrate the relationship between occupational exposure to UVR and the incidence of KC (9–12). As anticipated, MM is also associated with UVR exposure, but especially intermittent solar radiation exposure, and in particular in early life, and accordingly the relation with occupational solar UVR exposure is considered less conclusive, even if some recent studies suggested a possible association of specific MM subtypes, such as lentigo maligna melanoma (LMM), with chronic lifetime sun damage (5, 13).

### Current Status of the Management of the Occupational Solar UVR Risk

Millions of outdoor workers worldwide are exposed solar UVR during a major part of their working time. Despite this circumstance, this work-related risk factor is in many countries still not formally recognized by occupational safety and health (OSH) directives and regulations, and no specific occupational exposure limit values are officially available as a standard (14). A possible result of this inhomogeneous and scant recognition of the occupational risk is far from adequate implementation of fundamental preventive interventions for outdoor workers, as indicated by the large number of studies reporting high levels of individual UVR exposure at work (14, 15) and the inadequate adoption of sun-protective habits and behaviors by these workers (16). Among the negative consequences of this under-recognition of occupational risks associated with UV exposure, there is a lack of reporting of the cases, a lack of evidence on the effectiveness of health surveillance programs and screenings for the high-risk groups of OWs, a lack of compensation for cancer cases and a lack of political awareness to this increasing occupational health problem (13, 17, 18).

### Collective and Individual Interventions for the Prevention of the Risk of UV-Induced Skin Cancers Among Outdoor Workers

Preventive interventions can be related to primary, secondary, and tertiary prevention. Primary prevention includes any preventive action aimed at reducing the incidence of cancer in humans (19). Considering the primary prevention of UV-induced skin cancers among outdoor workers, the strategies to be adopted can be on a collective and, if needed, also on an individual basis (20). First of all, it should be noted that primary prevention of occupational risks at the workplace could not be referred only to a company level, but it can be included in a wider approach, related to governmental and institutional preventive actions and policies, and the predisposition of specific norms, guidelines and preventive campaigns (18, 20). At the workplace, the first step of primary prevention includes the establishment of an adequate risk assessment process, to be reviewed and updated regularly. Based on the results of the risk evaluation, appropriate actions can be taken, including (but not limited to) technical measures as e.g., roofing of outdoor workplaces, use of panels and glasses to reduce solar UVR, and organizational measures as e.g., the organization of indoor work-breaks or, when not possible, breaks in shaded places, and the reduction of the exposure during the middle hours of the day (20).

Other important collective measures for the prevention of the occupational risk are the information of the workers, including e.g., the provision of informative materials like leaflets, signs or phone-messages, and the performance of specific educational training activities, including sun-safety trainings and skin cancers prevention trainings. These initiatives, and in particular those involving the educational training of the workers, can increase the knowledge and the appropriate perception of the occupational solar UVR risk, and they are considered fundamental for the prevention of skin cancers in outdoor workers (14, 16, 20).

On an individual basis, primary prevention of occupational risks consists of providing appropriate Personal Protective Equipment (PPE) to the workers. To reduce excessive solar UVR exposure, the individual protections available include: (1) sunglasses meeting adequate standards with appropriate solar UVR filtering large lenses, adhering to the face and large temples; (2) clothes made of UVR filtering fabrics, with long-sleeved shirts and trousers; (3) appropriate headgears as broad-brimmed helmets when required, or hats, possibly supplied with sun shields and a neck guard (19, 20). Moreover, other individual preventive protections are sunscreens, even if they cannot be considered PPE: appropriate sunscreens must filter both UV-A and UV-B rays, with a Sun Protection Factor (SPF) of at least 30, but better 50 or more, based on the photo-type and the UV-index. Sunscreens need to be water-resistant, easily applicable on the body and have to be frequently re-applied. To reach the protection level indicated by the SPF, the quantities to be applied are about 2 mg/cm^2^ (20–22).

Secondary prevention includes the methods that can lead to the detection of precancerous conditions or cancers at an early stage (23). The two cornerstones of secondary prevention are screening and early diagnosis: in the workplaces, probably the most important measure of secondary prevention is the occupational health surveillance (HS) of the workers judged to be at increased risk of adverse effects, being exposed to relevant levels of solar UVR. HS aims at the prevention and the early diagnosis of UV-related adverse effects, with specific attention to subjects with conditions possibly determining a particular susceptibility to the risk (e.g., a fair skin photo-type). Moreover, HS usually includes periodic medical examinations of the workers from trained occupational health professionals, requiring, in case, supplementary health controls to be decided on an individual basis and the involvement of other medical specialists, such as dermatologists (13, 14, 18).

Finally, also tertiary prevention should be mentioned, even if it intervenes when the adverse effects are already manifested. Interventions in this field include the medical and occupational rehabilitation of the workers with UV-related skin cancers after the therapies and are aimed at ensuring a safe return to work, with full recovery from the disease and an adequate quality of life, as well as compensations for the occupational diseases diagnosed and properly notified to the authorities (18, 20).

### Objective of the Systematic Review

The systematic review aims to fill a relevant gap in the scientific literature, evaluating the effectiveness of the available preventive interventions, as e.g., the ones listed in the previous sub-section, conducted in outdoor workplaces to reduce the solar UVR related risk of the exposed workers, with the final purpose of the prevention of UV-induced skin cancers among outdoor workers according to the definitions provided in the “IARC Handbooks of Cancer Prevention” (19, 23). A few other systematic reviews have been published on similar topics (16, 24–28), but none of these focused on interventional studies specifically in the broader context as defined by the framework outlined by the preambles of the IARC Handbooks of Cancer Prevention (19, 23).

## Methods

### Protocol and Registration

The present protocol has been submitted to the International Prospective Register of Systematic Reviews (PROSPERO). The PROSPERO registration number is CRD42021251891. The current protocol follows the preferred reporting items for systematic reviews and meta-analysis protocols (PRISMA-P) (29) and subsequently the systematic review will be reported according to the respective preferred reporting items for systematic reviews and meta-analysis (PRISMA) statement (30). In accordance with PRISMA-P this protocol provides the rationale for the systematic review, as well as the pre-planned methodological and analytic approach (29). The review process will start after the final definition of the protocol and all the phases are planned to be completed within the subsequent twelve-months.

### Eligibility Criteria

We will consider eligible all the studies evaluating the effectiveness of interventions to reduce exposure to occupational solar UVR and the risk of skin cancers among outdoor workers. Our overall P.I.C.O. question is as follows:

Population = outdoor workers exposed to solar UVR targeted with preventive interventions aimed at reducing their skin cancer risk.

Intervention = preventive interventions, including primary and secondary prevention based on collective and individual measures addressed to outdoor workers, as:

a) Political and/or institutional initiatives, as the establishment of preventive actions to reduce the risk of UV-induced skin cancers among outdoor workers at a regional/national level.
b) Collective workplace interventions, including technical and organizational measures to reduce solar UVR exposure and the skin cancers risk.
c) Personal sun-safety information and training for the workers, including also specific campaigns aimed at raising awareness of the risk of skin cancers linked with solar UVR exposure, and of the importance of adopting adequate UVR protective behaviors, and of using appropriate personal protection.

Comparison = outdoor workers exposed to solar UVR for whom no preventive interventions aimed at reducing their skin cancer risk has been established.

Outcome = primary and secondary outcomes of the studies included in the systematic review are the following:

a) Primary outcome: effectiveness of the interventions in reducing the incidence of UV-induced skin cancers (SC) among outdoor workers, which are mainly KC, but considering also possible effects on malignant melanoma incidence in solar UV-exposed workers.

b) Secondary outcomes, considered as indirect measures of a reduced SC risk for outdoor workers: effectiveness of the interventions in implementing/improving/increasing the considered preventive measure(s)/protection(s), or reducing the incidence in case of adverse health effects, depending on the specific outcome as listed in the secondary outcomes.

#### Inclusion and Exclusion Criteria

Our target population is the working-age population, excluding child labor and unpaid domestic workers. We will consider outdoor workers (e.g., construction workers, farmers, gardeners, lifeguards, fishermen, and others) exposed to solar UVR in the workplace as the target population.

We will include studies of any publication year investigating the effects of different workplace sun-safety interventions and their effects on the reduction of occupational exposure to solar UVR and the incidence of skin cancers in exposed workers and on other secondary outcomes as listed below in the secondary outcomes. Studies written in any of the languages spoken by the Authors (i.e., English, French, Italian, German, Portuguese, and Spanish) will be included. Only human interventional studies with an adequate group for comparison (i.e., outdoor workers for whom the same interventions were not provided) will be considered. The types of study designs that will be included are interventional studies, both randomized and non-randomized, as well as observational studies, including case-control and cohort studies. Cross-sectional studies, as well as case-series studies and case-reports and publications without original data (e.g., reviews, letters to the editor, and editorials) will be excluded.

#### Types of Outcome Measures

The overall outcome of this systematic review is to assess the effectiveness of sun-safety interventions at work for the prevention of occupational skin cancers.

We refer to the definitions of “effectiveness” and interventions for primary and secondary prevention as reported respectively in the “IARC Handbooks of Cancer Prevention: preamble for primary interventions” (19) and in the “IARC Handbooks of Cancer Prevention: preamble for secondary interventions” (23).

##### Primary Outcome

The primary outcome of this systematic review is to assess the effectiveness of sun-safety interventions at work to reduce the incidence of occupational skin cancers, which are mainly KC, including basal cell carcinoma and squamous cell carcinoma, ICD-10 code C44, but considering also possible effects on cutaneous malignant melanoma incidence in solar UV-exposed workers, ICD-10 code C43.

##### Secondary Outcomes

The secondary outcomes considered are the following:

a) The reduction of the incidence of other solar UV-related skin diseases, e.g., sunburns, photo-aging, actinic keratosis, which are positively associated with an increased SC risk.
b) The improvement of the knowledge and of the risk perception of outdoor workers and employers concerning occupational solar UVR exposure and related health risks.
c) The improvement of the solar UVR exposure habits and protective behaviors of outdoor workers,
d) The implementation of new specific collective preventive interventions in the workplaces, including technical and/or organizational measures to reduce solar UVR exposure.
e) The improvement of the current preventive practices at a political/institutional level, e.g., the establishment of new preventive actions or campaigns aimed at reducing the SC risk for outdoor workers.

It should be noted that points (c), (d) and (e) represent both “interventions” possibly applied in specific studies, as well as secondary outcomes, to be evaluated after an appropriate follow-up, of an intervention aimed at reducing the SC risk for outdoor workers.

### Information Sources and Search Strategy

The electronic databases searched for this systematic review will be PubMed MEDLINE, EMBASE, and Scopus.

The search strategy is being developed on PubMed MEDLINE by two co-authors and will then be revised and tested by the co-authors and a Medical Librarian Expert. We are designing the search strategy to specifically address the study's objectives, including detailed terms related to PICO criteria and aiming not to miss any important studies in the field. After validation of the search, we will translate it for EMBASE and Scopus.

We will search also gray literature for publicly available materials, including reports and databases from recognized international organizations active in the field of cancer prevention (e.g., World Health Organization, International Labour Office, etc.), government agencies, and institutions of national occupational insurance systems, such as INAIL (Italy) or DGUV (Germany).

Finally, we will also include a hand search of the reference lists of previous reviews (forward and backward citation tracking) and eligible articles. Scientific articles written in any of the languages spoken by the Authors will be included. There will be no restrictions on the publication period. The expected date of the last update of the literature search is 31st of December of 2021.

### Study Records

#### Data Management

The citations retrieved from the three electronic databases will be downloaded as Research Information Systems (RIS) files and imported into a literature administration software (e.g., EndNote X9, Zotero, Mendeley, etc) and into the software used for facilitating the study selection process (e.g., Covidence, Rayyan, etc.), with automatic identification and exclusion of the duplicates upon importation.

#### Selection Process

The results of the literature searches will be imported into the identified software(s) for the initial screening, after the removal of the duplicates.

The selection of the potentially eligible studies will rigorously follow the pre-determined inclusion and exclusion criteria outlined above.

The first step of the selection process includes the screening of titles and abstracts, which will be performed independently by at least two reviewers, while third reviewers not having participated in this screening phase will solve any conflicts of inclusion.

After the initial screening, the full texts of potentially eligible studies will then be examined by at least two reviewers. Also, in this case, eventual conflicts will be solved by third reviewers not involved in the screening, while any other discrepancies at all stages of study selection will be resolved through discussion and consensus among the Authors' group. Results of the screening process will be presented in a PRISMA flow chart (29, 30).

#### Data Extraction Process

Each study will be double-reviewed and data will be independently extracted in pre-defined tables reporting all the relevant information (e.g., study ID, title, country, study setting, population, participant's characteristics, type of study, starting date, ending date, method of recruiting participants, the total number of participants, type of intervention, intervention goal, intervention assessment, outcome data, conflicts of interests). The data extraction forms will then be checked by a third Author for accuracy. Discrepancies between the data extractors will be discussed until reaching a consensus. A detailed data extraction sheet is being developed specifically for this study and will be piloted in a minimum of four studies.

### Quality Assessment of Individual Studies

We will assess the risk of bias of all the individual studies included in the systematic review. The assessment will be independently performed by two Authors and possible conflicts solved by a third Author. We will base our assessment on published tools for the assessment of the risk of bias in the studies, considering the IARC Preambles, and in particular, the points presented in the sub-chapter “Study quality and informativeness” (19, 23).We will use the Cochrane collaboration group tools ROBINS-I and RoB2, respectively for non-randomized and randomized studies (31). The overall risk of bias of the individual studies will be rated as low, moderate, serious, critical or with no information for non-randomized studies using ROBINS-I while low, some concerns or high for randomized studies based on an evaluation with the RoB2 tool.

### Data Synthesis

We will provide a qualitative narrative synthesis of the aggregated results of the included studies, supported by forest plots and categorized by type of preventive intervention(s) provided to the workers and type of primary and secondary outcomes measured to evaluate the effectiveness of the intervention(s). The results will be summarized in tables containing the year, country, population and participants (outdoor workers), type of intervention and outcome(s), and the main relevant results (e.g., incidence rates, relative risks, etc.), unadjusted and adjusted, in this case with the reporting of the considered confounders. A descriptive synthesis of the findings from the included studies, structured from the interventions and outcomes details, will be provided. We will also perform subgroup analysis, considering the specific categories of outdoor workers (e.g., construction workers, fishermen, farmers, etc.), their ethnic/cultural background if available and the geographic area where the studies have been conducted. Whenever enough data (>2 estimates) available, we will conduct meta-analyses separately for estimates of the effectiveness of the intervention on the specific outcome. When we will find two or more studies with eligible effectiveness of intervention estimate, two Authors will independently investigate the heterogeneity of the studies in terms of types of studies, participants (including country, sex, age, and industrial sector or occupation), risk factor exposure, intervention, comparator and outcomes. If we will judge two or more studies for the relevant combination of country, sex, and age groups, or a combination thereof, to be sufficiently homogenous to potentially be combined quantitatively using quantitative meta-analysis, then we will test the statistical heterogeneity of the studies using the I^2^ statistic. When the studies will be found to be sufficiently homogenous statistically, we will pool the risk ratios of the studies in a quantitative meta-analysis, using the inverse variance method with a random-effects model to account for cross-study heterogeneity. If quantitative synthesis will not be feasible, then we will synthesize the study findings and identify the estimates taking into account the overall evidence by considering the informativeness of the studies and the results of the risk of bias assessment.

## Discussion

Solar UVR-induced occupational skin cancers are an extremely relevant issue for outdoor workers (14, 17, 18), and while some general evidence on a positive effect in limiting the occupational solar UVR exposure of these workers is available (16, 25, 26, 28), precise and valid data on the effectiveness of interventional studies for the reduction of the incidence of SC in solar UV exposed workers are still lacking. In particular, this systematic review will follow the principles defined by the IARC in its Handbooks of Cancer Prevention (19, 23). Accordingly, we will investigate the effectiveness of the interventions defined in the IARC preambles, and consequently, we will be able of providing reliable indications for the actual reduction of skin cancers incidence in outdoor workers.

### Strength and Limitations

Considering methodological aspects, the systematic review aims to follow a rigorous method for all the steps of the process, including study selection, data extraction, quality assessment, and reporting of the results, following internationally recognized tools, like those of the PRISMA and Cochrane research groups (30, 31). The main strength of our review will be, as mentioned above, the full adherence with the statements expressed by the IARC for the definitions of the effectiveness of the interventions for cancers' prevention (19, 23).

Unfortunately, we expect a probably low number of studies directly evaluating the primary outcome defined in the present protocol, i.e., the effectiveness in reducing the incidence of occupational SC in outdoor workers, and therefore we may need to focus on secondary outcomes as indirect indications of the decrease in SC occurrence: this will be most likely the main limitation of our systematic review.

We also expect to have a relevant number of studies rated with a poor quality assessment, according to the fact that we expect a majority of non-randomized studies, in which it would be more difficult to evaluate the effectiveness of the interventions due to the presence of various biases.

### Dissemination

The systematic review will be submitted for publication to an international peer-reviewed scientific journal. Systematic review's summaries will be further presented in the form of structure scientific communications and articles for journals and national or international conferences.

## Ethics Statement

Ethical review and approval was not required for the study on human participants in accordance with the local legislation and institutional requirements. Written informed consent for participation was not required for this study in accordance with the national legislation and the institutional requirements.

## Author Contributions

KS, SJ, TL, AM, and FG: conceptualization. MS, TL, KS, CS, and MR: methodology. MS, TL, MR, CS, AM: software. TL, FG, KS, SJ: resources. AM, MS, CS, and MR: data curation and writing—original draft preparation. TL, KS, SJ, and FG: writing—review and editing and supervision. All authors have read and agreed to the published version of the manuscript.

## Funding

The costs for open-access publication will be covered by the European COST action OMEGA-NET (CA16216).

## Conflict of Interest

The authors declare that the research was conducted in the absence of any commercial or financial relationships that could be construed as a potential conflict of interest.

## Publisher's Note

All claims expressed in this article are solely those of the authors and do not necessarily represent those of their affiliated organizations, or those of the publisher, the editors and the reviewers. Any product that may be evaluated in this article, or claim that may be made by its manufacturer, is not guaranteed or endorsed by the publisher.
